# Lower Mesophotic Coral Communities (60-125 m Depth) of the Northern Great Barrier Reef and Coral Sea

**DOI:** 10.1371/journal.pone.0170336

**Published:** 2017-02-01

**Authors:** Norbert Englebert, Pim Bongaerts, Paul R. Muir, Kyra B. Hay, Michel Pichon, Ove Hoegh-Guldberg

**Affiliations:** 1 Global Change Institute, The University of Queensland, St Lucia, QLD, Australia; 2 Australian Research Council Centre of Excellence for Coral Reef Studies, The University of Queensland, St Lucia, Queensland, Australia; 3 School of Biological Sciences, The University of Queensland, St Lucia, QLD, Australia; 4 Queensland Museum, Townsville, QLD, Australia; Department of Agriculture and Water Resources, AUSTRALIA

## Abstract

Mesophotic coral ecosystems in the Indo-Pacific remain relatively unexplored, particularly at lower mesophotic depths (≥60 m), despite their potentially large spatial extent. Here, we used a remotely operated vehicle to conduct a qualitative assessment of the zooxanthellate coral community at lower mesophotic depths (60–125 m) at 10 different locations in the Great Barrier Reef Marine Park and the Coral Sea Commonwealth Marine Reserve. Lower mesophotic coral communities were present at all 10 locations, with zooxanthellate scleractinian corals extending down to ~100 metres on walls and ~125 m on steep slopes. Lower mesophotic coral communities were most diverse in the 60–80 m zone, while at depths of ≥100 m the coral community consisted almost exclusively of the genus *Leptoseris*. Collections of coral specimens (n = 213) between 60 and 125 m depth confirmed the presence of at least 29 different species belonging to 18 genera, including several potential new species and geographic/depth range extensions. Overall, this study highlights that lower mesophotic coral ecosystems are likely to be ubiquitous features on the outer reefs of the Great Barrier Reef and atolls of the Coral Sea, and harbour a generic and species richness of corals that is much higher than thus far reported. Further research efforts are urgently required to better understand and manage these ecosystems as part of the Great Barrier Reef Marine Park and Coral Sea Commonwealth Marine Reserve.

## Introduction

Mesophotic coral ecosystems (MCEs) are found at intermediate depths (30 to ~100–150 m depth), and are characterized by the presence of scleractinian corals that (like their shallow-water counterparts) associate with dinoflagellates from the genus *Symbiodinium* (or “zooxanthellae”) [1]. Large areas of MCEs have been identified in the Indo-Pacific [2–4] and Western Atlantic (e.g. [5–7]), demonstrating that these ecosystems constitute a significant proportion of overall coral reef habitat [8]. However, most of the research efforts have concentrated on upper mesophotic depths (30–60 m), given the potential of communities at these depths to provide a refuge against disturbances (e.g. warm-water bleaching and tropical storm damage) and act as a subsequent source of reproduction for damaged shallow reefs (given the substantial overlap in community structure) [9]. In contrast, studies on lower mesophotic depths (>60 m) remain relatively sparse (but see [10–13]), in part because their greater depth makes them harder to access. However, recent evidence from the Caribbean demonstrates that the lower mesophotic zone can host distinct, deep-specialist communities and represent a separate entity within the coral reef ecosystem [14].

For the world’s largest coral reef ecosystem, the Great Barrier Reef (GBR), studies have also focused primarily on the upper mesophotic zone, reporting diverse assemblages of both zooxanthellate scleractinian and octocorals [4, 15–17]. Yet, we still know surprisingly little about the presence and composition of lower MCEs, despite the original descriptions of lower mesophotic communities in this region dating back to the 1980s. Manned submersibles were used to confirm the presence of (zooxanthellate) coral-dominated communities at lower mesophotic depths at Myrmidon Reef and Ribbon Reef No. 5 (GBR; [18]) and Osprey Reef (Coral Sea; [19]). Since then, only a single survey has evaluated coral communities at lower mesophotic depths, reporting depth zonation patterns at four sites on the GBR [4, 20]. At these locations, either a dominance of azooxanthellate octocorals or a transitionary community containing both zooxanthellate and azooxanthellate taxa (60–75 m depth) was found to occur [20]. Nonetheless, there is extensive potential habitat (i.e. hard-substrate slopes in clear oceanic waters) for zooxanthellate scleractinian corals at lower mesophotic depths on the shelf edge reefs of the northern GBR (which extend over ~700 km) [21] and the steep walls of the numerous Coral Sea atolls (that rise out of deep oceanic waters) [15]. Yet, these lower mesophotic depths remain largely unexplored, in part due to the strict occupational safety constraints in scientific diving in this region. Consequently, confirmation of the extent of lower MCEs has been hampered by the requirement for manned or remotely controlled underwater vehicles.

Zooxanthellate coral communities can extend to great depths, with zooxanthellate scleractinian corals observed in Hawaii and Johnston Atoll down to 153 and 165 m depth, respectively [11, 22]. Light availability is one of the key factors determining the vertical distribution of zooxanthellate scleractinian corals and is, to a large extent, predictable over depth due to the light attenuation characteristics of the water column [23]. In optically clear waters, light can penetrate to great depths with ~1% of the surface light irradiance reaching down to around 100 m in Hawaii [12]. The deepest observed limits for zooxanthellate corals are generally associated with locations with the highest optical water quality, which supports the hypothesis that light availability determines the depth distribution limits of zooxanthellate corals [23]. However, other factors such as temperature, sedimentation and reef structure might also play either a direct or indirect (by modulating the light availability) role in determining the lower depth limits of zooxanthellate corals [14, 24]; yet these factors and their interaction remain less well understood. While a recent study revealed that zooxanthellate corals can extend to at least 125 m depth on the GBR and Coral Sea atolls [25], it remains unclear to what extent this lower depth limit of zooxanthellate corals varies across north-east Australia, and how it relates to local environmental conditions.

Based on the community structure data from elsewhere in the Indo-Pacific, the zooxanthellate scleractinian coral genus *Leptoseris* appears to be a key member of the lower mesophotic zone [23]. Additional coral genera *Montipora*, *Porites* and *Pachyseris* have been reported to occur in this depth zone in the Central Pacific Ocean [12, 26], yet were rarely found to be dominant or abundant below 60 m [but see 3]. Initial assessments of mesophotic coral biodiversity on the GBR [17, 27] revealed several new geographic and depth range extensions, however only a very small number of scleractinian corals from lower mesophotic depths (>60 m) were recovered in these studies. Thus far, only 8 zooxanthellate scleractinian coral genera and 14 different species have been recorded at lower mesophotic depths (≥ 60 m) on the GBR and in the Coral Sea [15, 17–19, 27]. The low coral diversity reported to date could be due to the very limited sampling effort undertaken at lower mesophotic depths [27] and the lack of studies focusing specifically on the more diverse region of north-east Australia [28]. Despite the challenge of sampling at these depths, such studies are required to improve our understanding of the overall biodiversity on the GBR, and to determine the extent of unique biodiversity associated with the potentially extensive deep-water ecosystems.

Here, we summarise qualitative assessments of lower mesophotic coral ecosystems carried out using a remotely operated vehicle (ROV) during five targeted research expeditions. In total, 15 different sites (representing 10 different reef locations) in the Great Barrier Reef Marine Park and the Coral Sea Commonwealth Marine Reserve were assessed, representing several degrees of latitude. The main aims of this study were to (1) identify the presence and lower depth limits of lower MCEs in north-east Australia; (2) assess broad patterns of zooxanthellate scleractinian coral community structure (at genus level) across the lower mesophotic depth gradient; and (3) evaluate whether these lower mesophotic depths harbour a low diversity of (zooxanthellate) scleractinian coral species.

## Materials and Methods

### Study sites

**Fig 1:**
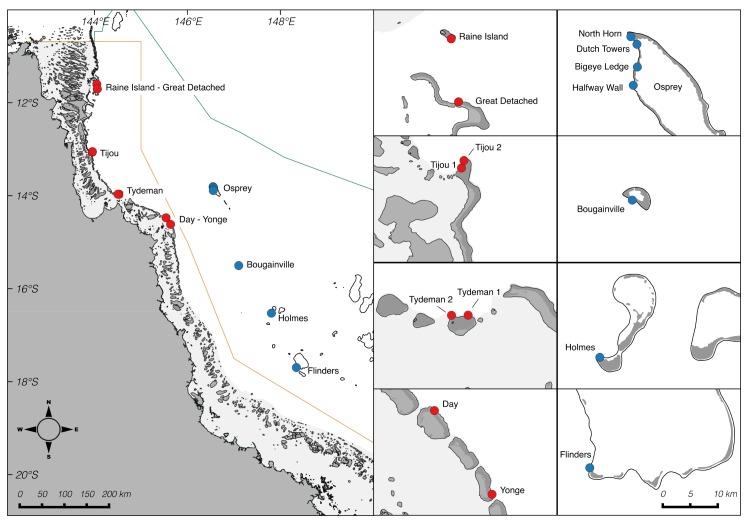
Map of survey locations. Survey locations in the Great Barrier Reef Marine Park (red dots) and the Coral Sea Commonwealth Marine Reserve (blue dots). Map produced with data files courtesy of Great Barrier Reef Marine Park Authority and “www.deepreef.org” under the Creative Commons Attribution Licence (CCAL) CC BY 4.0.

Lower mesophotic depths (60–125 m) were surveyed at a total of 15 different sites across 10 locations (Fig 1) in north-east Australian waters during five expeditions of the “Catlin Seaview Survey” carried out between September and December 2012, in November 2013, and in November-December 2014. Surveys and coral collections were conducted under permits from the Department of the Environment (018-CZRS-1207626-01, 018-RRRW-131031-01, AU-COM2012-151 and AU-COM2013-226) and from the Great Barrier Reef Marine Park Authority (G12/35281.1 and G14/37294.1). In the Great Barrier Reef Marine Park, we surveyed one site each at Raine Island, Great Detached Reef, Day Reef and Yonge Reef, and two sites each at Tijou Reef and Tydeman Reef (Fig 1). In the Coral Sea Commonwealth Marine Reserve, we surveyed four sites at Osprey Reef and one site each at Bougainville Reef, Holmes Reef and Flinders Reef (Table 1). Locations were chosen across a broad geographical range and, for many locations only a single site could be accessed due to sea-time limitations (full assessment of a site equated to a full day at sea). Individual sites were selected based on (and consequently biased towards) steep bathymetric profiles to allow for a relatively shallow anchorage with close-access to deep water for ROV operations.

**Table 1:** Details of surveyed locations including the site names, survey dates and coordinates (latitude and longitude).

Reef location	Site name	Survey date	Latitude	Longitude
Raine Island	Raine	01-Dec-14	S11° 36' 04.6"	E144° 02' 55.4"
Great Detached Reef	Great Detached	11-Dec-12	S11° 42' 14.8"	E144° 03' 38.8"
Tijou Reef	Tijou 1	14-Dec-12	S13° 03' 52.0"	E143° 57' 02.4"
	Tijou 2	29-Nov-14	S13° 03' 07.1"	E143° 57' 17.6" *
Tydeman Reef	Tydeman 1	28-Nov-14	S13° 58' 03.9"	E144° 31' 52.6"
	Tydeman 2	28-Nov-14	S13° 58' 04.8"	E144° 30' 11.8"
Day Reef	Day	18-Dec-12	S14° 28' 27.5"	E145° 32' 20.6"
Yonge Reef	Yonge	19-Dec-12	S14° 36' 58.6"	E145° 38' 12.6"
Osprey Reef	Osprey North Horn (NH)	29-Oct-12	S13° 48' 27.0"	E146° 33' 03.2"
	Osprey Dutch Towers (DT)	26/27-Oct-12	S13° 49' 13.2"	E146° 33' 40.4"
	Osprey Bigeye Ledge (BL)	23-Oct-12	S13° 51' 30.4"	E146° 33' 43.0"
	Osprey Halfway Wall (HW)	29-Oct-12	S13° 53' 21.3"	E146° 33' 18.1"
Bougainville Reef	Bougainville	23-Nov-13	S15° 30' 09.9"	E147° 06' 09.2"
Holmes Reef	Holmes	17/18-Sep-12	S16° 31' 33.0"	E147° 48' 23.9"
Flinders Reef	Flinders	21-Sep-12	S17° 41' 41.0"	E148° 20' 36.8"

* Approximate location.

### Benthic video transects and analyses

Benthic video transects were carried out using a vLBV-300 ROV (SeaBotix, San Diego USA) at 7 depth intervals (every 10 m) in the lower mesophotic zone between 60–120 m. Qualitative transects documenting the benthic community were conducted for 5–20 minutes at each depth, to assess coral community structure, bathymetric features and substratum composition. Shorter excursions were made at several sites to a maximum depth of ~130 m. The main ROV camera recorded in standard-definition (640 x 480 pixels) with an information overlay with depth, time and heading, and a second camera recorded in high-definition (1980 x 1024 pixels; used for analyses) and was time-synchronized with the main camera. A third camera with 10x optical zoom was frequently used to verify coral identifications *in situ*. For the bathymetric profile the following four categories were used: gentle slope (~10–30°), moderate slope (~45°), steep slope (~60°) or wall (~80–90°). The dominant substratum classes were scored *sensu* Bridge et al. [20]: sand/gravel/rubble (SGR), sediment-covered limestone (SCL) and limestone (L). Observed coral colonies were identified to genus level, as accurate species-level identification (which often requires microscopic examination of corallites for genera like *Montipora*) was usually not possible from the video footage. In addition, it was difficult at times to discriminate between some members of the genera *Montipora* and *Porites*, between members of the family Fungiidae, and between members of the genera previously ascribed to the family Pectiniidae (i.e. *Echinophyllia*, *Mycedium*, *Oxypora* and *Echinomorpha*). These were therefore classified as, respectively, *Montipora*/*Porites*, Fungiidae and “Pectiniidae”. Given that travelled distance was not consistent across depths and a proportion of the corals within the surveys could not be identified from video, representation of the dataset was simplified to presence/absence data for each coral category (genus or family as described above). Colony sizes were categorized as small (<20 cm), medium (20–50 cm) and large (>50 cm), using the ROV manipulator as a size reference. Basic clustering and statistical analyses were carried out using the “vegan” package in R (v3.3.1). Hierarchical clustering (hclust) and analysis of variance (adonis) between transects was assessed using a Jaccard dissimilarity matrix of presence/absence data (only transects with ≥ 10 observations were considered). The difference in generic diversity between the main depth range categories (60–80 m and 90–120 m) was assessed using a Mann-Whitney test.

### Temperature measurements

Temperature loggers (HOBO Pro v2 model U22-001; Onset Computer Corp., Bourne USA) were calibrated (at the Australian Institute of Marine Science, Townsville, Australia) and deployed at three sites on the northern GBR (Great Detached, Tijou 1 and Yonge) and at four study sites in the Coral Sea (Osprey Bigeye Ledge, Osprey Halfway Wall, Holmes and Flinders). Temperature loggers were deployed at depths of 10, 20 and 40 m (using SCUBA), and 60, 80 and 100 m (using the ROV) at each study site, and recorded water temperature at 5-minute intervals over a period of 22–48 hours from which averages were calculated.

### Coral specimen collections

Coral specimens were collected at several study sites as a means of checking video-based identifications and for identification to species level. A total of 213 coral colonies were sampled from 60 to 125 m depth using the ROV manipulator. Coral samples were tagged, photographed, sampled for DNA analyses and the remaining skeleton prepared for laboratory identification by bleaching for 24–72 hours in a sodium hypochlorite solution (house-hold bleach diluted in fresh-water) followed by rinsing in freshwater and drying in sunlight. Identifications of the bleached coral skeletal samples were conducted by Carden Wallace, Paul Muir and Michel Pichon at the Queensland Museum (QM; in Townsville) and representative specimens have been registered in the QM State Collection under numbers: G68156, G68392, G69875, G69880, G69908, G69943, G71132-G71158. These specimens and the matching video footage of the *in situ* coral colonies were used as a basis for genus identification based solely on video footage.

## Results

### Bathymetric profiles, substrate composition and temperatures

**Fig 2:**
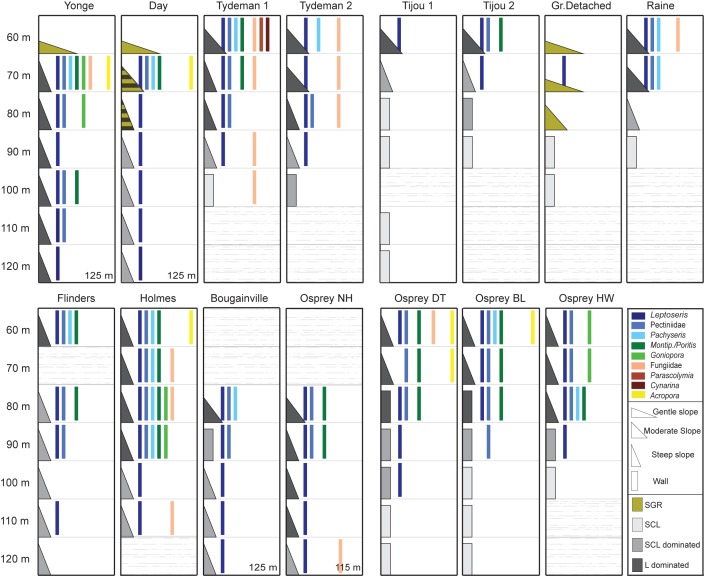
Presence/absence diagram of coral genera over depth across 15 survey sites on the Great Barrier Reef (top row) and Coral Sea (bottom row). Colours indicate different growth forms (blues = plating, greens = encrusting/plating, browns = solitary/free-living and yellow = branching) and shades indicate different taxonomic groups. For the benthic substrate, steepness is indicated with different shapes and dominant substrate type is indicated by colour (SGR = sand/gravel/rubble; SCL = sediment-covered limestone; SCL dominated = sediment-covered limestone dominated with some exposed limestone present; L dominated = exposed limestone dominated, but some sediment-covered limestone present). Grey horizontal “snow” means no transect performed at those depths.

**Fig 3:**
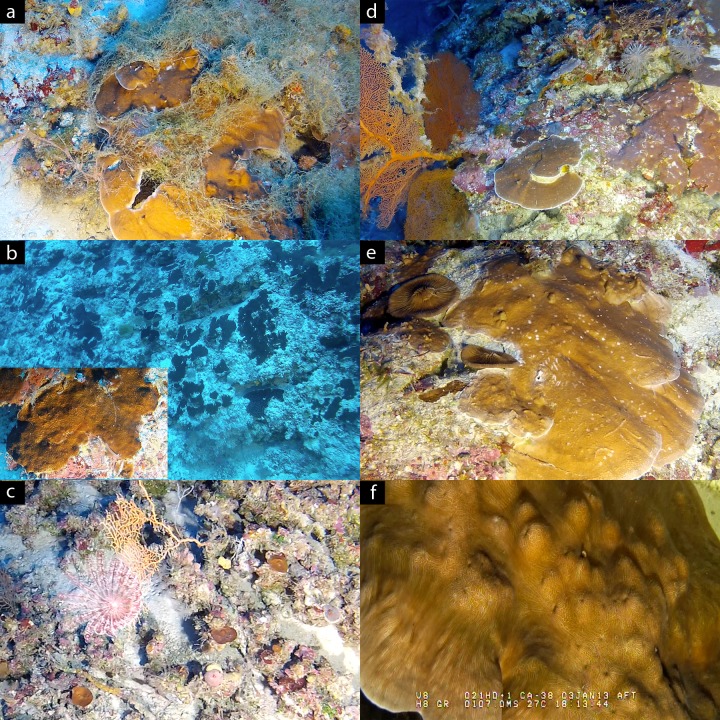
Video stills from benthic video transects. (a) Green macroalgae (genus *Cladophora*) covering a *Montipora* colony at Tydeman Reef at 70 m depth. (b) Monostand of fragmented *Goniopora* encountered at Osprey Reef HW around 70 m depth, interrupted by small sand patches and sediment-covered limestone. Close-up of colony inserted in left bottom corner. (c) Deep *Leptoseris* community consisting of small (<20 cm) individual corals at Yonge Reef at 120 m depth. (d) Limestone outcrop at 70 m extending down to ~90 m depth supports large plating and encrusting corals at Holmes Reef. (e). *Montipora* coral with two free-living fungids at Holmes Reef (80 m depth). (f) *Leptoseris hawaiiensis* coral at Bougainville Reef at 107 m depth (with ROV video overlay).

Great Barrier Reef–Two distinct bathymetric profiles were observed (Fig 2) on the surveyed outer reef locations on the northern GBR: (1) a plateau consisting of sand, gravel and rubble dominated by *Halimeda* macroalgae (gently sloping; ~10–30°) turning into a steep slope around 70–80 m (Yonge and Day) or near-vertical wall around 90 m depth (Great Detached), and (2) a moderate slope (~45°) of limestone covered in varying degrees with sediment that became steeper with depth and eventually turned into a near-vertical wall between 80–100 m depth (Raine, Tijou, Tydeman). *Halimeda* macroalgae were only found to be dominant in the first profile (down to depths of 90 m) and two sites at Tydeman had a high abundance of *Cladophora* down to 75 m depth (Fig 3A). Limestone ridges and ledges were present at most sites, but were particularly pronounced at Yonge and Day below 70 m, with large sand channels in between these formations.

Coral Sea—The seven study sites in the Coral Sea all exhibited a steep slope (~60°) (starting from a ledge at around ~30–40 m depth) down to a very narrow terrace or ledge somewhere between ~60–70 m depth, which then continued as a steep slope (~60°; Flinders, Holmes, Bougainville, Osprey North Horn) or a near-vertical wall (~80–90°; Osprey: Dutch Towers, Bigeye Ledge, Halfway Wall) down to at least 120 m (Fig 2). Bougainville and Osprey North Horn were characterized by a more moderately sloping profile (~45°) at around 80 m, where sediment accumulated around several large limestone protrusions with no defined sand channels. Large limestone formations and ledges (with large sand channels in between) were particularly pronounced at Holmes down to ~90 m depth.

**Fig 4:**
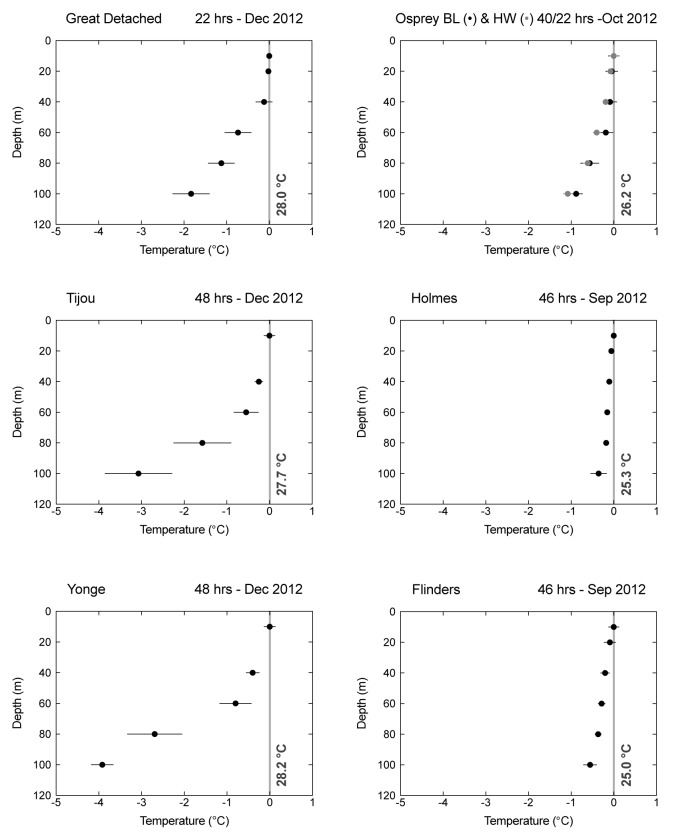
Short-term temperatures over a 10–100 m depth range for 7 of the study sites. Average temperatures (°C ± SD—indicated with arrow bars) at 10, 20, 40, 60, 80 and 100 m depth expressed relative to 10 m mean temperatures. Note the different measurement durations (22–48 hrs) and different months in which the temperature data was collected.

Temperature—The average seawater temperature at shallow depths (10 m) ranged between 27.7 and 28.2°C for our GBR sites (Great Detached, Tijou 1 and Yonge; December 2012). In the Coral Sea, average temperature at 10 m ranged from 26.1 to 26.3°C at the Osprey sites (October 2012), and were 25.0°C and 25.3°C, respectively, for Flinders and Holmes (September 2012). Mean temperatures only decreased slightly over depth (≤1.1°C between 10 and 100 m depth) at the Coral Sea sites. Although recorded at a different time of the year, on the GBR these differences were greater, and ranged from ~1.8 to 3.9°C between 10 and 100 m depth. Temperature variability also increased with depth below 60 m at the GBR sites (expressed as SD; Fig 4), whereas such a pronounced increase in variability with depth was not observed for the Coral Sea sites.

### Coral community structure

Across the lower mesophotic zone (60–120 m), the most abundant and diverse zooxanthellate scleractinian coral communities were observed in the shallowest depth range of 60–80 m, with zooxanthellate scleractinian corals present at all 15 sites (Fig 2). In this depth range, 347 out of 524 observed coral colonies were confidently identified from the video footage according to our classification scheme (i.e. mostly representing genus-level identification), and represented members of 13 different genera. Coral colonies of the genera *Leptoseris*, *Pachyseris*, *Montipora/Porites* and belonging to the “Pectiniidae” were the most frequently observed corals in the depth range of 60–80 m, and were present at 10 to 15 sites.

*Leptoseris* colonies consisted exclusively of small- (<20 cm) to medium-sized (20–50 cm) plates, whereas colonies of *Pachyseris*, *Montipora/Porites* and “Pectiniidae” showed a mixture of plating and encrusting morphologies with colony sizes ranging from small (<20 cm) to very large (>1 m). Branching *Acropora* spp. were observed at 4 locations (Day, Yonge, Holmes and two Osprey sites) between 60 and 70 m depth (previously reported in [20]). Several individual colonies of the usually solitary and generally free-living corals from the family Fungiidae were found at three GBR locations (Raine, Tydeman and Yonge) and two Coral Sea locations (Holmes and Osprey) between 60 and 80 m depth. Several large *Goniopora* colonies (>2 m diameter) were observed at Holmes around 80 m depth, whereas the *Goniopora* colonies at Yonge were small- to medium-sized (<50 cm). At one of the Osprey sites, an extensive monospecific stand of fragmented *Goniopora* (>20 m diameter) was encountered on the steep reef slope around 70 m, interrupted by small sand patches and sediment-covered limestone (Fig 3B).

Zooxanthellate scleractinian coral colonies were observed to at least 90 m depth at 11 of our 15 study sites (the exceptions being the most northern GBR sites: Raine, Great Detached and the two sites at Tijou). At these depths (90–125 m), the coral community was dominated by small (<20 cm) *Leptoseris* colonies. On the GBR, at Day and Yonge, there was a substantial presence of these small *Leptoseris* colonies even at depths between 110 and 125 m (n = 90 colonies counted; Fig 3C), whereas in the Coral Sea their presence was much sparser, but with colonies still extending to 100 m (western Osprey sites), 110 m (Holmes and Flinders), 115 m (Osprey North Horn) and 125 m depth (Bougainville). Corals of the genera *Montipora*/*Porites* and from the family “Pectiniidae” were rare in the 90–100 m depth range and, except for one large (>50 cm) cf. *Echinophyllia* colony at 110 m depth at Yonge Reef, were not observed below 100 m. Furthermore, several individual free-living corals (family Fungiidae) were encountered in the depth range of 90–100 m at Tydeman 1 and around 110 m at Holmes and Osprey North Horn. Although benthic cover by zooxanthellate corals was very low in the depth range of 90–125 m across our study sites, we encountered several larger (~40–120 cm) plating and encrusting zooxanthellate scleractinian corals on a vast limestone outcrop at ~90 m at Holmes Reef (Fig 3D and 3E) (*Leptoseris*, “Pectiniidae”, *Montipora/Porites*, *Pachyseris* and *Goniopora*), and along the steep reef slopes at Bougainville (Fig 3F) and Osprey North Horn (*Leptoseris*, “Pectiniidae”, and *Pachyseris* or *Montipora/Porites* respectively).

Hierarchical clustering confirmed the general partitioning of transects at depths above and below 90 m (S1 Fig), and generic richness was significantly higher in the shallower depth range (Mann-Whitney W = 158.5, p-value = 0.005303). Analysis of variance confirmed significant differences between transects in relation to depth (adonis p-value = 0.001) and region (adonis p-value = 0.036).

### Species-level richness

The coral specimens collected from 60 to 125 m depth represented a total of 29 different species, belonging to 18 different genera and 8 families. Of these species 23 could be confidently identified down to species level, 4 provisionally identified (“cf.”), and 2 identified as potential new species (to be followed up on in a subsequent study) (Table 2). Four of these species were members of the genus *Acropora* previously reported by Muir et al. [17]. Two additional zooxanthellate colonies for which no specimens were collected were confidently identified from the video footage: *Parascolymia vitiensis* and *Cynarina lacrymalis*. The vast majority of these species were collected and observed around 60–65 m depth (23 out of 31). Observations of the following species represent depth-limit extensions: *Bernardpora stutchburyi*, *Cycloseris vaughani*, *Leptoseris glabra*, *Leptoseris mycetoseroides*, *Lithophyllon undulatum*, *Montipora aequituberculata*, *Oxypora glabra*, *Oxypora lacera*, *Podabacia crustacea*, *Porites rus*, *Parascolymia vitiensis* and *Stylocoeniella guentheri*. A specimen of the coral species *Craterastrea levis* was collected from 60 m at Osprey Reef and this represents the first occurrence record of this species in north-east Australia.

**Table 2:** Zooxanthellate scleractinian coral species collected at lower mesophotic depths (60–125 m) on the Great Barrier Reef (GBR) and in the Coral Sea (CS).

Species	Max. collection depth	Collection location(s)
*Acropora aculeus*^1^	60 m	Osprey (CS)
*Acropora echinata*^1^	60 m	Osprey (CS)
*Acropora cf*. *granulosa*^1^	73 m	Day (GBR)
*Acropora speciosa*^1^	60 m	Osprey (CS)
*Alveopora cf*. *tizardi*	60 m	Osprey (CS)
*Bernardpora stutchburyi*^2^	60 m	Osprey (CS)
*Coscinaraea* sp.	60 m	Osprey (CS)
*Craterastrea levis*^3^	60 m	Osprey (CS)
*Cycloseris vaughani*^2^	60 m	Osprey (CS)
*Cynarina lacrymalis*^4^	60 m	Tydeman (GBR)
*Cyphastrea* sp.	60 m	Osprey (CS)
*cf*. *Echinophyllia* sp.	101 m	Yonge (GBR)
*Echinomorpha nishihirai*	82 m	Osprey (CS)
*Leptoseris fragilis*	80 m	Tydeman (GBR)
*Leptoseris cf fragilis*	124 m	Day (GBR)
*Leptoseris glabra*^2^	60 m	Tijou (GBR), Osprey (CS)
*Leptoseris hawaiiensis*	125 m	Bougainville (CS)
*Leptoseris mycetoseroides*^2^	60 m	Osprey (CS)
*Leptoseris scabra*	80 m	Holmes (CS), Osprey (CS)
*Lithophyllon undulatum*^2^	60 m	Osprey (CS)
*Montipora aequituberculata*^2^	60 m	Osprey (CS)
*Montipora millepora*	60 m	Osprey (CS)
*Mycedium elephantotus*	60 m	Tijou (GBR), Osprey (CS)
*Oxypora glabra*^2^	60 m	Tijou (GBR), Flinders (CS)
*Oxypora lacera*^2^	64 m	Osprey (CS)
*Pachyseris speciosa*	80 m	Holmes (CS), Osprey (CS)
*Parascolymia vitiensis*^2^^,^ ^4^	60 m	Tydeman (GBR)
*Podabacia crustacea*^2^	60 m	Osprey (CS)
*Porites rus*^2^	65 m	Osprey (CS)
*Seriatopora hystrix*	60 m	Osprey (CS)
*Stylocoeniella guenther*^2^	60 m	Osprey (CS)

^1^ Previously recorded in Muir et al. [17].

^2^ New depth record for Great Barrier Reef / Coral Sea.

^3^ New record of occurrence for Australia.

^4^ Species described from video observations.

## Discussion

Our study demonstrates the presence of lower mesophotic communities (60–125 m) harbouring zooxanthellate scleractinian coral at all the assessed locations, highlighting that these communities are likely dominant features throughout the outer reefs and atolls of the GBR and Coral Sea. Zooxanthellate scleractinian coral communities were observed to occur down to at least ~100 metres on walls and ~125 m on steep slopes in this region. Specimen collections confirmed that zooxanthellate diversity was substantially higher than reported previously [15, 17–19, 27], with 29 species from at least 18 different genera identified at lower mesophotic depths.

### Coral community structure at lower mesophotic depths

Although earlier surveys from the GBR and Coral Sea documented the presence of zooxanthellate scleractinian corals at lower mesophotic depths [15, 18–20, 27], we have demonstrated that such lower mesophotic coral communities are in fact common on the outer reefs and atolls off north-east Australia, given that all 15 surveyed sites harboured zooxanthellate corals at ≥60 m depth and about half of these sites harboured corals down to at least 100 m depth. Across our study sites, the lower mesophotic zooxanthellate coral community was dominated by the genera *Leptoseris*, *Montipora/Porites*, *Pachyseris* and genera previously ascribed to the *“*Pectiniidae” family, confirming previous observations from other parts of the Indo-Pacific that these predominantly plating/encrusting species dominate the lower mesophotic zone [12, 22, 29, 30].

Community structure was observed to be highly variable between and within locations, ranging from the occurrence of a single genus to members of >10 different genera, which is broadly similar to other parts of the Indo-Pacific (e.g. [3, 12]). Diversity was highest between 60 and 80 m depth, with the lowest reaches of the mesophotic zone (100–120 m) dominated almost exclusively by the genus *Leptoseris*. These observations are again similar to other parts of the Indo-Pacific [23] and reflect the unique ability of this genus to withstand extremely low light conditions [31–34]. Several members of the family Fungiidae were observed between 100 and 120 m depth in the Coral Sea, and while alive and well-pigmented, it cannot be excluded that these have recently tumbled down the slope from shallower depths. Besides the zooxanthellate scleractinian coral genera that are dominant in the lower mesophotic zone, colonies belonging to the genera *Acropora* and *Goniopora* were occasionally observed. Although *Acropora* is generally considered to be a “shallow-water” genus, a recent study by Muir et al. [17] demonstrated that *Acropora* spp. can be relatively abundant in the upper mesophotic zone (<50 m). Although much rarer in the lower mesophotic zone, individual colonies of *Acropora* spp. were observed at four locations with steep slopes between 60–70 m depth, with the deepest *Acropora* colony (*cf*. *A*. *granulosa*) recorded at 73 m at Day Reef [17].

Species of the genus *Goniopora* can form extensive beds on shallow inner reef slopes on the GBR (e.g. [28, 35]), although individual colonies of *Goniopora djboutiensis* have been reported to occur down to 58 m on the central GBR [27]. In the present study, we observed *Goniopora* corals in the lower mesophotic down to 90 m depth (Holmes), and noted that these can even form rather extensive monospecific stands (Osprey HW; 70 m depth). This was the only observed instance of a large monospecific stand in the lower mesophotic zone, with the scleractinian coral community normally consisting of sparse individual colonies (particularly below 90 m depth). High cover communities at lower mesophotic depths, such as the *Leptoseris* beds in the Hawaiian Archipelago [12] and *Agaricia* beds in the southern Caribbean, [36] were not observed at any of the sites. Given photographic evidence (dating back several decades) of such communities on the central GBR [16], this could potentially be related to the bias in site selection targeting steep slopes. Given the ubiquitous presence of sediment across sites, local bathymetric features were a strong determinant in the presence of scleractinian corals as protrusions and ledges offered suitable hard substratum. Such features were particularly abundant at the study sites on Yonge (70 m) and Holmes (80 m), which also harboured a high generic richness and large colony sizes.

### Lower depth limits of zooxanthellate coral communities

Lower depth limits of zooxanthellate coral communities were variable between sites and locations. There was, however, an apparent pattern related to the geomorphology of the sites. All sites with a continuously sloping reef profile harboured corals down to 110–125 m, whereas those with a near-vertical wall had much shallower depth limits of 80–100 m. Although light availability ultimately determines the maximum depth at which zooxanthellate corals can occur, near-vertical walls can greatly reduce the available irradiance [37] and provide a more challenging substratum for scleractinian corals to establish and grow on [38]. This was particularly apparent when comparing between sites at Osprey, where incident irradiance and light attenuation are presumably similar across sites, but strong differences in lower depth limits were observed that may be the result of differences in reef profiles (North Horn versus other sites).

Depth limits were relatively “shallow” (60–70 m depth; Fig 2) at the northern-most sites of the GBR (Tijou, Great Detached and Raine), where zooxanthellate corals stopped occurring at depths before the near-vertical wall of this particular reef profile started. The narrower shelf [39] and major channels in the reef allow for major exchanges between inshore and oceanic waters (“Second Three-mile Opening” and the “Raine Island Entrance”) [40] at these locations, and appears to result in a higher light attenuation coefficient, with noticeably lower visibility observed both in the ROV footage and by divers during sampling.

Short-term temperature logger data from 4 sites in the Coral Sea showed that water temperatures only varied up to ~1°C from 10–100 m depth in spring (September-October), indicating that the water column was well-mixed. This is consistent with previous observations of strong vertical mixing throughout most of the year in the region [41], despite temperature fluctuations due to upwelling in the summer months [42]. The temperature measurements on the GBR were performed in the beginning of summer (December), with considerable temperature difference recorded over depth. The largest temperature differences (~4°C between 10 and 100 m) were observed for the site (Yonge Reef) with the lowest observed depth limit (125 m) of zooxanthellate corals, and temperature is therefore unlikely to be the limiting factor for coral distribution on the other two sites that have shallower coral depth limits (Tijou 1 and Great Detached). Nonetheless, strong temperature fluctuations at depth and/or long-term exposure to colder conditions could contribute to coral depth distribution over ecological timespans, but require assessment of temperatures over much longer time periods.

### Species richness at lower mesophotic depths

Indo-Pacific scleractinian coral biodiversity at lower mesophotic depths remains poorly understood, with most of our knowledge dating from primarily submersible surveys done several decades ago (e.g. [11, 19, 29, 30]) and a small number of recent studies (e.g. [12, 22, 43]). North-east Australia is probably one of the best-studied regions in terms of upper mesophotic coral biodiversity [4, 17], and initial efforts to obtain species richness estimates have been undertaken [27]. Here, we have demonstrated that the extremely low diversity previously reported at mesophotic depths is largely the result of the limited efforts at these depths so far and the strong bias towards visual surveys, as we documented the occurrence of at least 29 different scleractinian species (from 18 different genera). Our study increases the total number of reported species at lower mesophotic depths in eastern Australia from 14 (8 different genera) to 33 (18 different genera). Despite that the present study still represents a limited effort (in terms of ground covered and number of specimens collected) relative to the vast extent of mesophotic habitat predicted off north-east Australia [44], our results demonstrate that species richness in the lower mesophotic zone is much higher than initially reported.

Many of the identified coral species represent depth records for the region, extending the lower depth limits by 10 to 40 m for 12 of the recorded species (Table 2). Furthermore, one of the specimens was identified as *Craterastrea levis* [45] from 60 m depth at Osprey. This species has only previously been reported from the western Indian Ocean, Chagos Archipelago and the Red Sea [46–48], and represents the first published record of this species to occur in north-east Australia. This species is a typical depth-specialist, and may explain why it has not previously been recorded elsewhere in the south-west Pacific. Additionally, two specimens were collected that are likely to represent new species, and are currently undergoing further analysis.

## Conclusions

This study highlights the ubiquitous presence of lower MCEs on the outer reefs of the GBR and atolls of the Coral Sea. In addition, we found that the depth limits of tropical coral communities are highly variable in the region. Nonetheless, diverse zooxanthellate scleractinian coral communities were usually observed down to depths of around 80 m, with deeper sections dominated by sparser *Leptoseris* communities. The strong apparent role of reef bathymetry on the presence and composition of lower mesophotic communities highlights the potential of modelling studies to predict the distribution and abundance of these deep-water communities, which may be extensive in the region. The observed prevalence and relative diversity of scleractinian corals at lower mesophotic depths highlights the importance of studying these largely undocumented sections of the Great Barrier Reef Marine Park and the Coral Sea Commonwealth Marine Reserve. A better understanding is urgently required to allow for adequate consideration in future marine zoning by the respective management authorities.

## Supporting Information

S1 Fig Cluster dendrogram. Site name, depth and generic richness (in brackets) indicated per branch.
(PDF)
