# Supplementary figures and images for: Lower Mesophotic Coral Communities (60-125 m Depth) of the Northern Great Barrier Reef and Coral Sea
Source: PLoS One. 2017 Feb 1;12(2):e0170336. doi: 10.1371/journal.pone.0170336 (PMC5287465; doi:10.1371/journal.pone.0170336)

S1 Fig

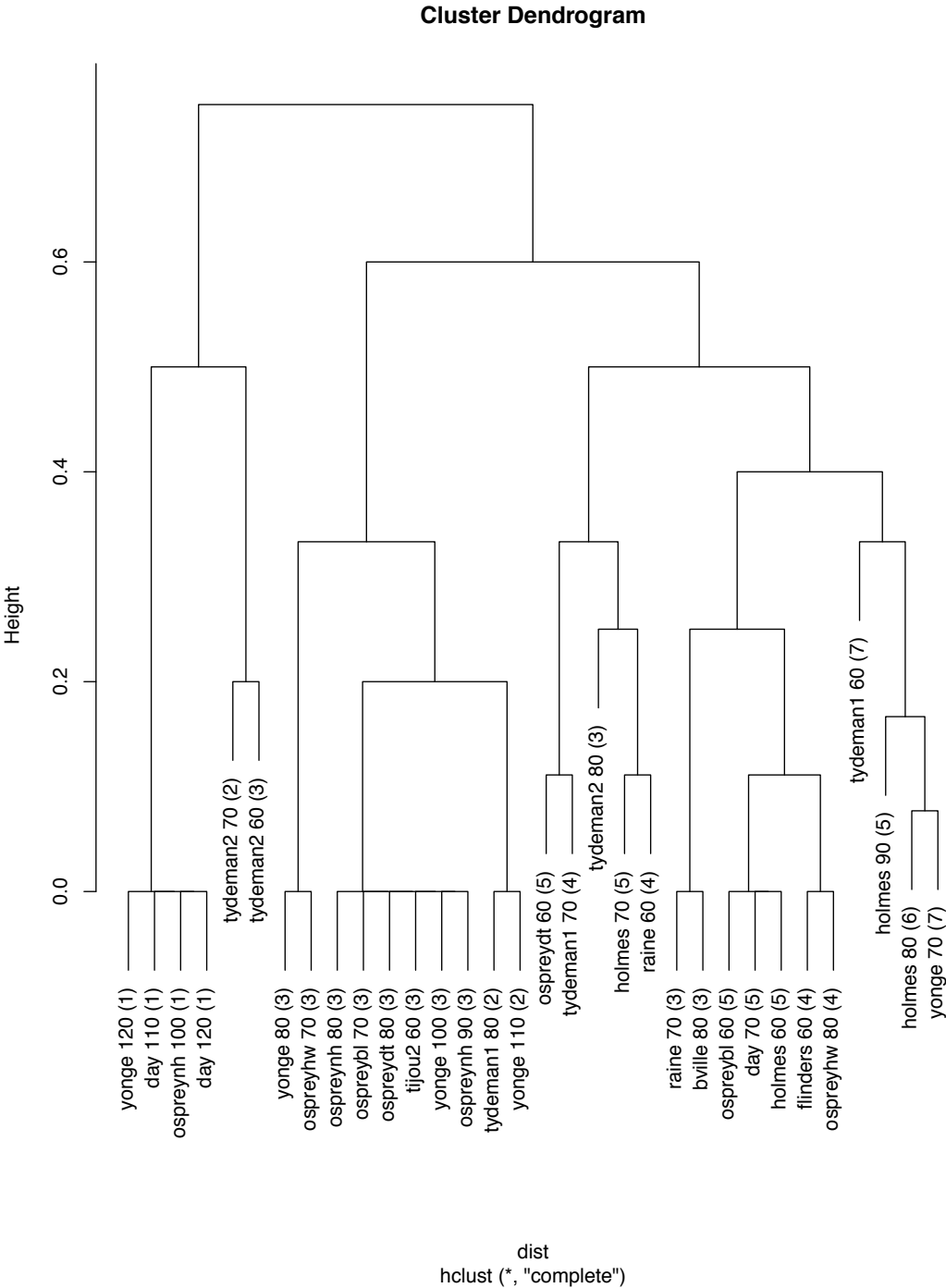

Supplement: S1 Fig — Site name, depth and generic richness (in brackets) indicated per branch. (PDF) [file pone.0170336.s001.pdf]
